# Partial shading by solar panels delays bloom, increases floral abundance during the late-season for pollinators in a dryland, agrivoltaic ecosystem

**DOI:** 10.1038/s41598-021-86756-4

**Published:** 2021-04-02

**Authors:** Maggie Graham, Serkan Ates, Andony P. Melathopoulos, Andrew R. Moldenke, Sandra J. DeBano, Lincoln R. Best, Chad W. Higgins

**Affiliations:** 1 Department of Biological and Ecological Engineering, Oregon State University, Corvallis, OR 97330 USA; 2 Department of Animal and Rangeland Sciences, Oregon State University, Corvallis, OR 97330 USA; 3 Department of Horticulture, Oregon State University, Corvallis, OR 97330 USA; 4 Department of Botany and Plant Pathology, Oregon State University, Corvallis, OR 97330 USA; 5 Department of Fisheries and Wildlife, Oregon State University, Hermiston Agricultural Research and Extension Center, Hermiston, OR 97838 USA

**Keywords:** Photovoltaics, Agroecology, Biodiversity, Ecosystem services, Restoration ecology, Urban ecology, Entomology, Light responses, Plant ecology, Plant physiology

## Abstract

Habitat for pollinators is declining worldwide, threatening the health of both wild and agricultural ecosystems. Photovoltaic solar energy installation is booming, frequently near agricultural lands, where the land underneath ground-mounted photovoltaic panels is traditionally unused. Some solar developers and agriculturalists in the United States are filling the solar understory with habitat for pollinating insects in efforts to maximize land-use efficiency in agricultural lands. However, the impact of the solar panel canopy on the understory pollinator-plant community is unknown. Here we investigated the effects of solar arrays on plant composition, bloom timing and foraging behavior of pollinators from June to September (after peak bloom) in full shade plots and partial shade plots under solar panels as well as in full sun plots (controls) outside of the solar panels. We found that floral abundance increased and bloom timing was delayed in the partial shade plots, which has the potential to benefit late-season foragers in water-limited ecosystems. Pollinator abundance, diversity, and richness were similar in full sun and partial shade plots, both greater than in full shade. Pollinator-flower visitation rates did not differ among treatments at this scale. This demonstrates that pollinators will use habitat under solar arrays, despite variations in community structure across shade gradients. We anticipate that these findings will inform local farmers and solar developers who manage solar understories, as well as agriculture and pollinator health advocates as they seek land for pollinator habitat restoration in target areas.

## Introduction

Pollinating insects are a cornerstone of natural and agricultural ecosystems, aiding in the reproduction of 75% of flowering plant species^1^ and 35% of crop species globally^2^. In the US, pollination services to agriculture are valued at $14 billion annually^3^. Habitat for pollinating insects is declining globally as a result of land use change, attributed in part to urbanization, agricultural intensification, and general land development^4^.

Changes in global climate can also cause shifts in habitat availability^5^. Global climate models predict increased aridity globally as the climate warms, and increased uncertainty around seasonal drought patterns^6, 7^. These impacts are especially visible in dryland ecosystems, where photosynthetic production is water-limited (sunlight is available in excess). Drylands account for 40% of land globally, and are defined by an Aridity Index (ratio of precipitation to potential evapotranspiration) of less than 0.65. This includes deserts, as well as temperate regions such as grasslands, savannahs, and Mediterranean ecosystems^6–8^. Changes in aridity, drought frequency, and drought severity, can cause shifts in temperature and that affect soil moisture, a key component of plant growth^7,9–11^. Drought conditions can impact floral abundance and decrease the available forage for pollinators, particularly later in the summer^9–11^.

Quality pollinator habitat requires access to soil, water, woody debris, and an abundance of nectar and pollen producing plants across the entire foraging season of individual pollinators or colonies^12^. Given emergence times and host-plant preference of pollinating insect taxa, habitat quality frequently depends on a diversity of flowering species that span a range of bloom shapes and bloom timings^13^. Honey bee and native bumble bee wintering success is strongly linked to nutrition, and in many regions of the Western US late-season bee taxa reproductive success is dependent on the availability of late-blooming plants^10,11,14^.

Solar photovoltaic (PV) installation in the US has increased by an average of 48% per year over the past decade, and current capacity is expected to double again over the next five years^15^. PV can be installed on a variety of surfaces including built structures, open land, or water. Sizes can range from small, backyard residential sites to multi-acre, utility-scale solar energy (USSE) systems. USSE installations can be a source of land cover change, and can impact ecosystem services, such as biodiversity, when installed in natural areas^16–22^. USSE has the potential to negatively impact biodiversity in wildland, desert ecosystems^19^, though impacts in temperate drylands and former agricultural lands are understudied.

When large, vegetated land surfaces are used for PV installations (e.g. agricultural fields, deserts, rangelands), the land is typically stripped of vegetation and graded^20^. A lower disturbance option exists to drill posts into the ground, though heavy machinery is still used which compacts soils and disturbs vegetation. After construction, the land is typically managed to limit plant growth since tall plants would block sunlight, decreasing energy generation. This management may include removing the existing vegetation, then covering with gravel or turf grass^22^. Rarely is the understory space managed for ecological conservation or used as productive agricultural land.

Installations in areas already impacted by human development, such as existing rooftops, parking lots, or degraded lands, can minimize the conversion of undeveloped land in land-limited environments^23^, and options exist for ecologically-synergistic, low-impact development^24^. One such option is agrivoltaics—a concept introduced in the 1980s^25^ where solar energy production is combined with agricultural production (dual-use) on the same land.

The concept of agrivoltaics has gained popularity in recent years as a means of creating low-impact solar energy development in agricultural communities^22^. In the United States, solar developers have begun to utilize the panel understory to promote both biodiversity and agricultural health by pairing PV with habitat for wild and managed pollinators^22,24^. Some states, such as Minnesota, North Carolina, Maryland, Vermont, and Virginia, have developed statewide guidelines and incentives to promote pollinator-focused solar installations^22^. In this practice, forage for pollinators is established as the solar array’s understory rather than the traditional turf grass or gravel. Some plantings focus exclusively on native species to prioritize restoration of native plant communities, others include a mix of native and non-native species.

Despite a recent surge in pollinator-focused solar installations, little is known about how solar panel canopies impact pollinators and the flowers they forage. Recent studies document the response of desert plants to PV in wildland ecosystems^19,26^, and crops such as pasture grasses^27^ and vegetables^28–30^ in agrivoltaic systems, yet none have addressed floral density or insect populations. Panel shading alters sunlight and soil moisture levels, creating a variety of microclimates within the solar understory^18,19,21,25–31^. Sunlight, water, and nutrients drive plant growth, which then impacts floral abundance and timing^32^. Floral abundance and localized shading then influence pollinator community structure^33–35^. However, the relationships among panel shading, plants, and pollinators have not been examined within a solar array.

To address this knowledge gap, we documented the species abundance, richness, and diversity of flowers and pollinators at a PV solar plant designed to provide habitat for pollinating insects and native plants. The objectives of our study were to (1) determine if pollinators would visit flowers in the solar array and (2) document the species abundance, richness, diversity, and composition of insect pollinator and plant communities across shade gradients (microclimates) within the solar array. We hypothesized that pollinators would visit flowers despite their location within the array, and that plant composition (as a result of species tolerance for shade and temperature) as well as pollinator composition (as a result of species tolerance for shade, temperature, and floral preference) would differ across shade gradients. Specifically, we hypothesized that partial shading by solar panels would create a microclimate that facilitates more abundant, more diverse flowers and pollinators compared to full sun (control) or full shade plots, particularly during the hot, dry months of July, August, and September.

## Methods

### Study location

**Figure 1:**
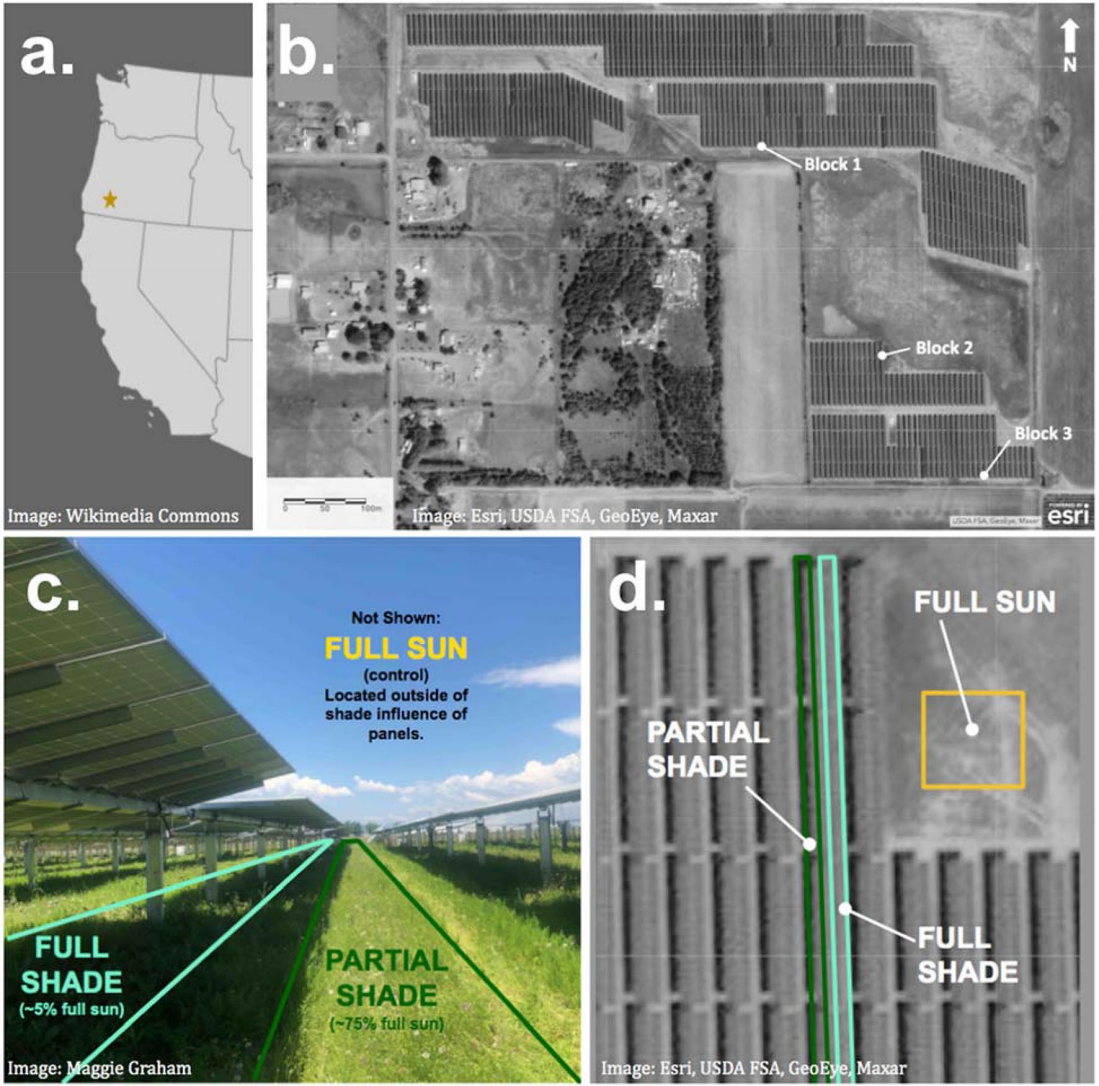
Site location and experimental design. The Eagle Point Solar Plant is (**a**) located in southern Oregon’s Rogue Valley. We established (**b**) three replicates within the site, each with three treatments (full sun as yellow, partial shade as green, full shade as blue), as shown in the (**c**) side view and (**d**) aerial view. Base imagery sources: (**a**) Wikimedia Commons 2019, (**b**,**d**) Esri 2021, USDA FSA, GeoEye, Maxar.

We conducted this study at the Eagle Point Solar Plant in Jackson County, Oregon (42°24′ N, 122°50′ W; Fig. 1). This 18 hectare (45 acre) site is located in the Rogue River Valley, west of the Cascade Mountains, and east of the Oregon Coast Range, within the traditional land of the Takelma peoples (Fig. 1a). The Rogue Valley is a predominantly agricultural region. Popular crops include wine grapes, pears, and other tree fruits. The site is bordered by agricultural fields (pears, hemp) and private residences. Permission to access the site was granted by Pine Gate Renewables, LLC.

The site soils are composed of Coker clay (33A), Padigan Clay (139A), and Phoenix Clay (141A) soils, all of which are Non-irrigated Class 4w soils^36^. At 412 m (1350 ft) of elevation, the site receives an average of 485 mm^37^ (19 in) of precipitation annually, and is considered a dryland, Mediterranean climate (2019 Aridity Index, 0.16^38^). The site is located in USDA plant hardiness zone 8b^39^.

In the fall of 2017, a 10 MW AC (13 MW DC) commercial solar generation facility was constructed on the site. The array consists of monocrystalline panels mounted on 3 m high racking with single axis tracking systems. Light sensors in the trackers cause the panels to rotate, following the sun throughout the day. Rows of panels are oriented along a north–south gradient, with panels tracking from east to west. Rows are spaced approximately 6 m on center. At the steepest angle of rotation (early morning, late evening), the lowest edge of the panel is approximately 1 m above the ground. When parallel with the ground (mid-day, sun overhead), the lowest edge of the panel is approximately 3 m above the ground.

Prior to solar development, the site was used primarily for cattle grazing^40^. The soils were highly compacted. Site vegetation primarily consisted of non-native rhizomatous grasses^40^. Small numbers of native and non-native forbs were also present at the site. Solar installation plans did not require massive grading, though some minor grading was prescribed for the site access road. Installation plans aimed to preserve existing vegetation outside of the required disturbance area. By nature of the installation process, some surface vegetation was removed, and surface soils were disturbed in areas where solar panels were installed. After installation, the site was prepared for restoration with native plants. In May 2018, clethodim was applied at 438 ml/ha (6 oz/ac) to portions of the site already occupied by native forbs, the remainder of the site was treated with glyphosate, applied at the manufacturer recommended rate. Additionally, bindweed (*Convolvulus arvensis*) was spot sprayed with glyphosate in June 2018. Manual removal of the highly invasive yellow starthistle (*Centaurea solstitialis*) occurred throughout the site in 2018 and 2019. In October 2018, the site was restored with a mix of native forbs and grasses, with the objective of providing habitat for both wild and managed pollinators^40^. The restoration species mix included a variety of annual and perennial forbs (Supplemental Material), many grown from seed collected onsite or nearby. Apart from *Festuca roemeri*, native grass species were not introduced during the initial planting to allow for continued grass-specific herbicide use, but were planned for future installation. Ongoing maintenance at the site consists of seasonal mowing, planting, and herbicide application, all part of the native plant restoration process. The site is not grazed or tilled, thus ground disturbance is minimal post-construction. Minimal to no woody debris is present on the site, though soil is abundant. A perennial water source is accessible to insects along the eastern edge of the property. An active apiary with 52 honeybee colonies is located along the southwest corner of the site, within flight distance of all survey locations (Supplementary Figure S1). Additional colonies are located on neighboring properties, and may be within flight range of the site.

### Experimental design

We collected observational data on pollinator and plant populations during seven sampling events in 2019, each spanning 2 days (June 11–12, July 2–3, July 14–15, July 30–31, August 13–14, August 27–28, and September 20–21). The study complied with all relevant institutional, national, and international guidelines and legislation. Sampling events started after peak bloom (late-April to mid-May) in early June, and continued through late September (“late-season”). We established the survey as a complete randomized block design with three replicates containing three 100 m^2^ treatment plots each (Fig. 1). Shade intensity was the treatment effect, and was determined by location within the solar array. Full shade (5% of total sunlight) plots were located directly underneath solar panel rows (Fig. 1c,d). Partial shade (75% of total sunlight) plots were located between solar panel rows, with the middle of the plot centered between the pilings of adjacent solar panel rows, which are approximately 6 m on center. Full sun (100% of total sunlight) plots, which served as controls, were located in open, unshaded areas still within the fenced property area (Fig. 1c,d). Effort was made to place full sun plots as close as possible to partial shade and full shade plots, with adjacent sides < 30 m apart.

We selected replicate locations based on the availability of suitable full sun plots which we located within the restored area, in areas not shaded by the solar panels (5 m from an east or west edge, 3 m from a north or south edge, and greater than 100 m^2^ in area). The individual width to length ratio of the 100 m^2^ full sun plots varied based on the configuration of available land and ongoing site maintenance activities (Supplementary Figure S1). For example, we had to shift the edge of the full sun plot in block 3 mid-season after a portion of the plot was mowed by site maintenance staff. The block centroid (central point between adjacent sides of treatment plots) for block 1 was located approximately 300 m from that of block 2 and approximately 500 m from block 3. The block centroid of block 2 was located approximately 200 m from that of block 3 (Fig. 1b). Differences among replicates was expected (ex. distance to apiary, soil/slope differences, etc.), which is why a complete randomized block design was chosen for the study design.

We collected climate data at three monitoring stations to provide context for the study, separating measurements by treatment when possible (Supplementary Figure S1). We collected net radiation (PYR Decagon Devices), air temperature (VP-3 Decagon Devices), and relative humidity (VP-3 Decagon Devices) at 15 min intervals at a height of 1.4 m. Soil moisture and soil temperature (GS-3 Decagon Devices) were also measured at 15 min intervals at a depth of 15 cm.

We used the line point intercept method to inventory botanical composition in plots^41^. In each plot, 100 data points were collected across five, 2 m transects at 10 cm intervals. In full shade and partial shade plots, transects ran from north to south (parallel to panels), and were positioned in the center of the plot, either directly underneath (full shade) or directly between (partial shade) rows of panels (Fig. 1c,d). In full sun plots, transects were in the center of rows 1.5 m apart. We selected the starting point of transects at random before each sample event. At each point intercept, we documented the species of the stem and the number of flowers in bloom per stem. Data points collected in each plot at each sampling event were added to determine a count of blooms per 100 m^2^ for each sample unit.

**Figure 2:**
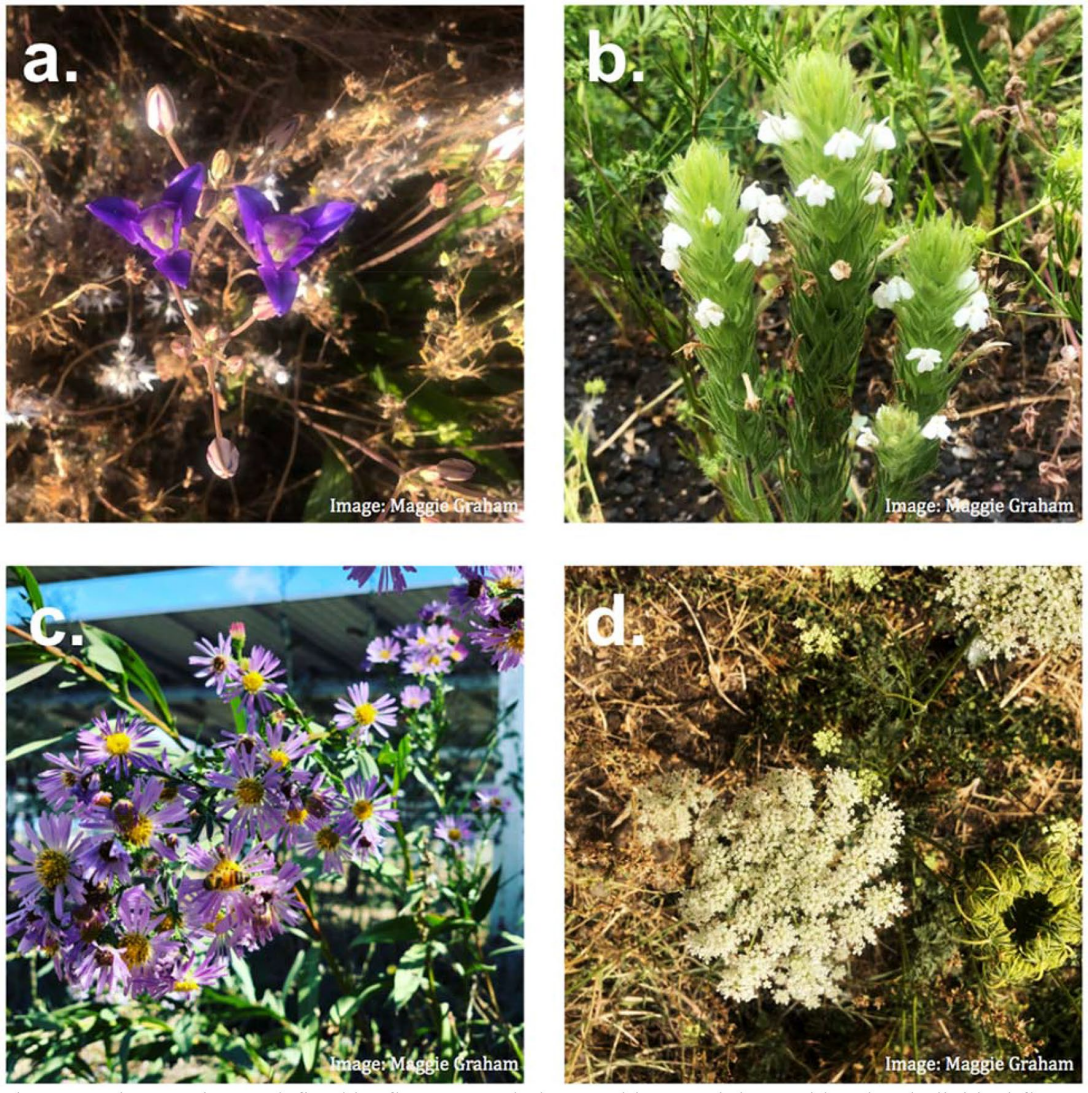
Bloom units are defined by flower morphology. A bloom unit is considered an individual flower for plants with (**a**) distinct, unclustered flowers or (**b**) stems of clustered flowers; a capitulum for plants with (**c**) distinct, composite flowers; and a flower head for plants with (**d**) multiple small, tight inflorescences.

Flower morphology, notably the number and arrangement of inflorescences in flowers, varies between plants. In this study, we are interested in the relative difference between treatment plots, not individual species. We defined “bloom” in a way that was practical for field survey of each plant. For plants with distinct, unclustered flowers (e.g. *Clarkia purpurea*, *Brodiaea elegans*), we considered each flower a bloom unit (Fig. 2a). For plants with stems of clustered flowers (e.g. *Castilleja tenuis*, *Vicia americana, Brassica nigra*, *Dipsacus* sp.), we considered individual flowers a bloom unit (Fig. 2b). For plants with distinct composite flowers (e.g. Asteraceae), we considered each capitulum a bloom unit (Fig. 2c). For plants with flowers composed of small, tight inflorescences (e.g. *Daucus carota*) it was not practical to distinguish between inflorescences, so we considered each flower head a bloom unit^42^ (Fig. 2d).

We collected insect specimens to inventory pollinating insect composition in plots. We used hand nets to survey insects visiting flowers in each plot during a 30 min sample event. We walked the plot continuously during this time, observing insects in consecutive 1 m^2^ zones. Specimens collected in each plot during each sample event were aggregated to determine a count of insects per 100 m^2^ per 30 min for each sample unit.

We sampled continuously between 9 am and 4 pm, on warm (> 16 °C), calm (< 20 km/h wind) days. Full sun and partial sun plots were surveyed when plots were unshaded. Unshaded surveys were not possible in full shade plots, which were surveyed when shaded. We collected all insects observed touching the reproductive parts of flowers, excluding individuals from the family *Miridae*, which were found in large quantities on stems, leaves, and flowers of some plants. After netting, we placed insects in ethyl acetate jars and froze for later identification. In the lab, we pinned, sexed, and identified specimens to species or the lowest taxonomic group possible. Taxonomists (Dr. Andy Moldenke and Lincoln R. Best) confirmed identifications and checked them with voucher specimens at the Oregon State Arthropod Collection, at Oregon State University in Corvallis, OR. An archived digital record of all specimens, including voucher material, is published to the Catalog of the Oregon State Arthropod Collection^43^.

### Statistical analysis

When conducting univariate analyses, we evaluated each sample unit (3 replicates × 3 treatment plots × 7 sample events = 63 total sample units) for differences in species abundance, species richness, species diversity, and visitation rate by performing a one-factor ANOVA (treatment) with repeated measures (sample event) and a blocking factor (replicate). We used a paired t-test with a Bonferroni correction to make pairwise comparisons of means. We conducted all univariate analyses in R version 3.6.1^44^ and used the vegan^45^ package to calculate species diversity. Our code is available in the Supplemental Material.

Before evaluating differences in species abundance, we logarithmically transformed counts of both blooms and insects (log(x + 1)) to improve normality and preserve extreme values^46^. We did not remove zero values (i.e., plots with no insects or no blooms), as these are important to the survey objectives. We defined species richness as the number of unique types (species or lowest taxonomic group possible) of individuals in a given sample unit^46^. We calculated species diversity for each sample unit using Shannon’s diversity index^46^. Visitation rate is defined as the ratio of insect abundance per minute, adjusted for the density of blooms^42^. This estimates insect use of floral resources relative to the number of resources available in each treatment, illuminating differences from factors other than floral density. We calculated visitation rate using (log (insects + 1)/(log (blooms + 1)) per 30 min per sample unit. Units without any insects and/or any flowers were assigned a value of zero.

For all univariate analyses, we evaluated the assumption of normality by plotting the quantiles of the model residuals against the quantiles of a Chi-square distribution, also called a Q–Q scatterplot. We evaluated the homogeneity of variances across treatments by creating box-whisker plots and confirming distribution was relatively equal for each tested variable.

We preformed multivariate analyses using PC-ORD Software version 7.07^47^. When conducting multivariate analyses, we aggregated species abundances from sample units by month (June, July, August/September) to form monthly sample units (3 replicates × 3 units × 3 months = 27 sample units). We then aggregated species-level abundances to higher taxonomic group-level abundances (Supplemental Material) to facilitate the analysis of community trends. The bloom group dataset contained total blooms per month for each replicate and treatment (27 monthly sample units × 13 taxonomic groups). The insect group dataset contained total insects per month for each replicate and treatment (27 monthly sample units × 13 taxonomic groups). The environmental dataset contained experimental design variables (27 monthly sample units × 4 variables) such as replicate, treatment, and month.

We used a nonmetric multidimensional scaling (NMS) ordination to compare the species community composition of monthly sample units. Ordination is a technique for summarizing complex, multivariate datasets, which are common in community ecology^46^. In an ordination, data points are arranged on axis according to how similar they are to each other. Points that are close on the graph are similar, points that are far are dissimilar^46^. We conducted NMS with relative Sorensen distances, 250 random starts (slow and thorough), and did not penalize ties. We used a randomization procedure to determine if solutions were more conclusive than expected by chance (P values), and calculated the percent variance explained by the model axes (R^2^ values). We used Pearson coefficients to determine significant (alpha = 0.05) relationships between taxa and ordination axes.

We used multiresponse permutation procedures (MRPPs) with relative Sorensen distances to evaluate the significance of differences in morphological group composition between groups of treatments and months (A-statistics, P values).

## Results

### Microclimate

**Figure 3:**
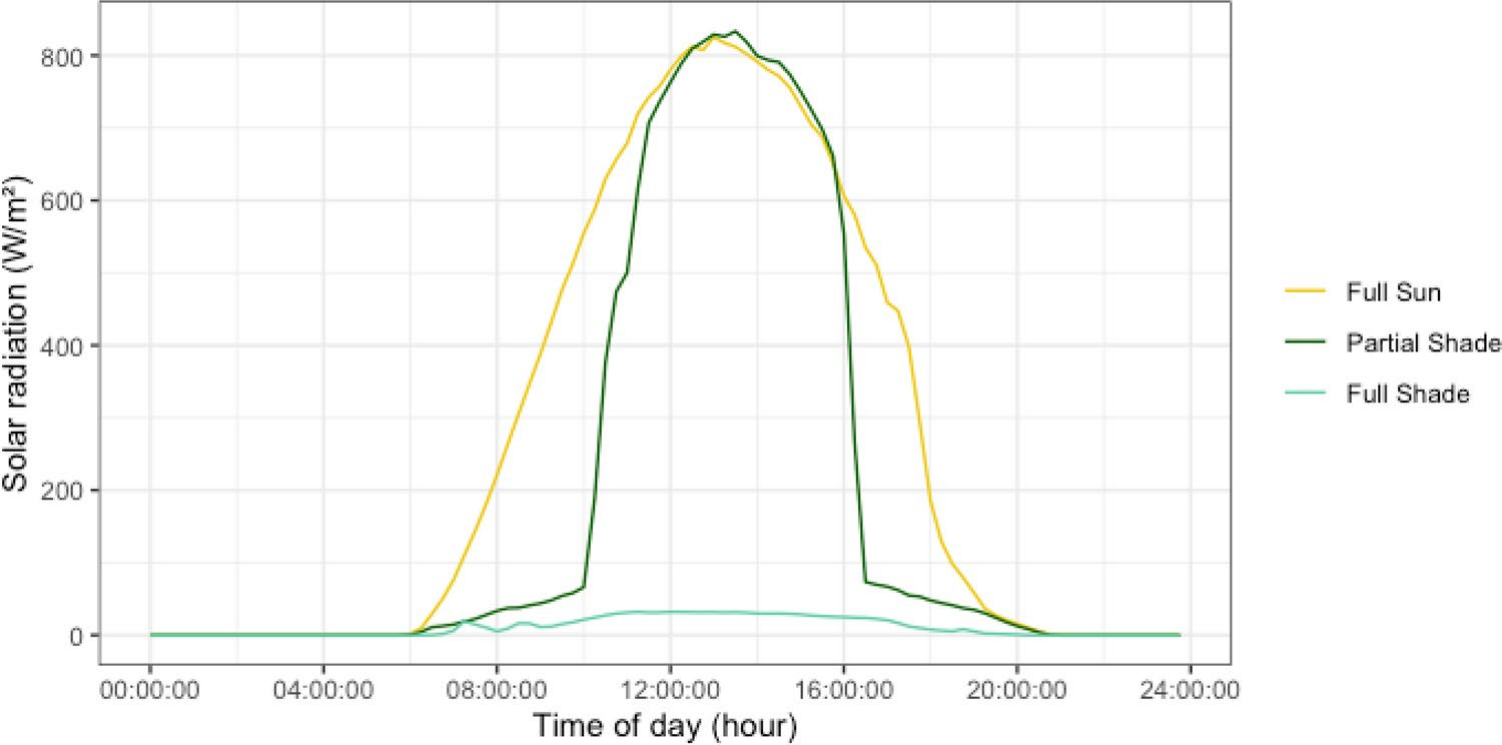
Average daily flux in solar radiation across the agrivoltaic system. Indicated by different color, for three treatments: full sun, partial shade and full shade.

Our unreplicated climate observations showed that solar panel shading alters the solar radiation, soil temperature, soil moisture, and vapor pressure deficit across treatments. From July to September, partial shade plots received approximately 75% of the solar radiation received by full sun plots, equivalent to an average of 3–4 fewer sun hours (roughly 10am to 4 pm versus 8am to 8 pm). The maximum radiation intensity was comparable around midday in full sun and partial shade plots (Fig. 3). In addition to reduced solar radiation, partial shade areas experienced reduced soil temperature, elevated soil moisture, and reduced vapor pressure deficit when compared to full sun plots (Supplementary Figure S2). Full shade plots received approximately 5% of the solar radiation received by full sun plots, and never received maximum radiation intensity (Fig. 3). In addition, full shade plots experienced reduced further soil temperature and when compared to both full sun and partial shade plots (Supplementary Figure S2). Soil moisture and vapor pressure deficit data is not available for full shade plots (Supplementary Figure S2).

### Floral resources

**Figure 4:**
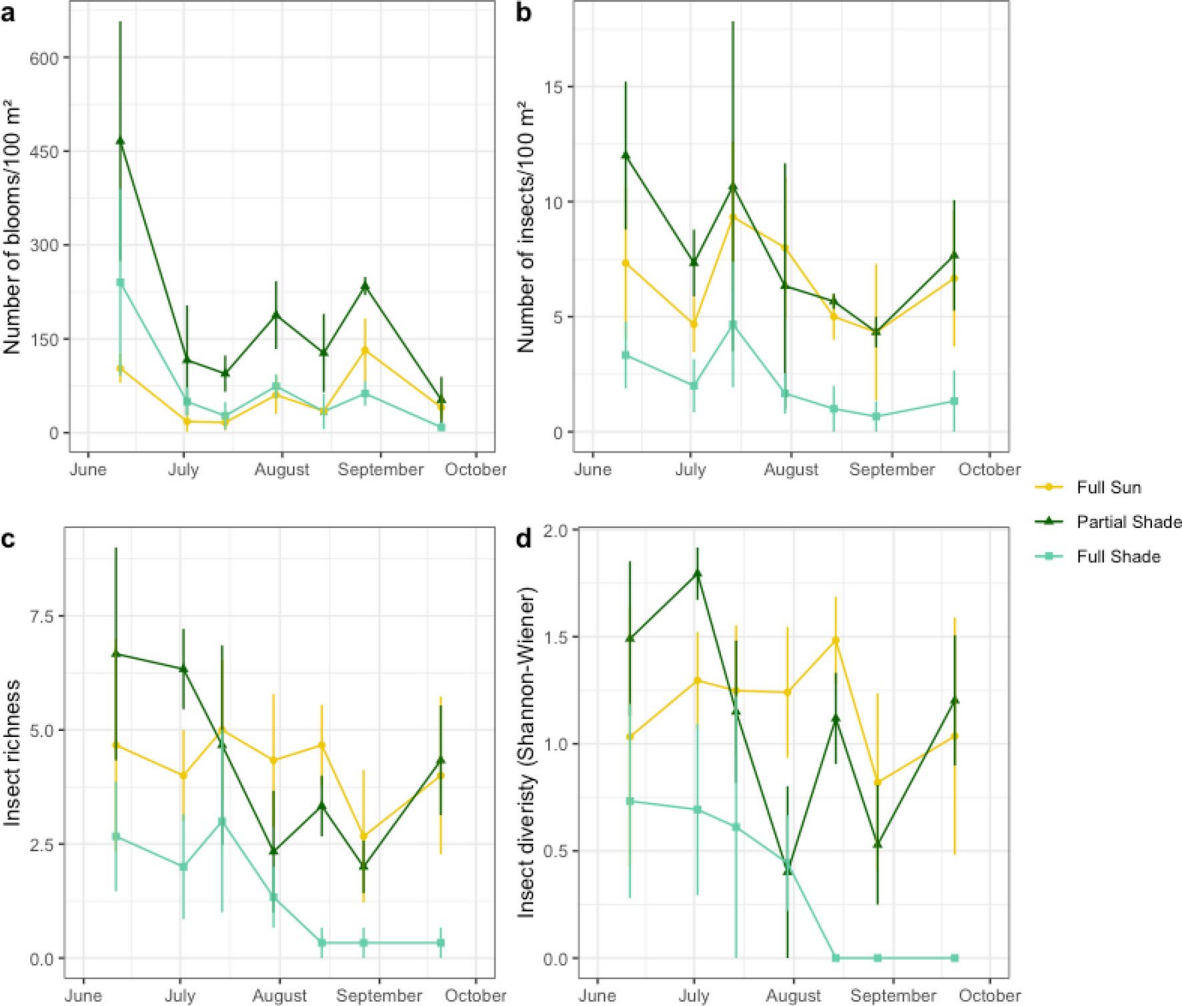
Plant and pollinator community populations over time by measurement type: (**a**) bloom abundance, (**b**) insect abundance, (**c**) insect richness, and (**d**) insect diversity. Each symbol represents the mean of 3 observed values, indicated by different color, for three treatments: (1) full sun (2) partial shade, and (3) full shade.

Over the course of the study, we collected 6,300 vegetation data points from 48 species of plants. Of these species, 26 were blooming at the time of survey. We counted a total of 6,543 bloom units on flowering stems. Floral abundance was greatest in partial shade plots, where we found 4% more blooms than in full sun (p = 0.008) and 4% more than in full shade plots (p = 0.019, Fig. 4a). Neither richness nor diversity of flowers differed among treatments (p = 0.11, p = 0.12 respectively). Floral abundance, richness, and diversity all differed temporally across the seven sampling dates (p = 0.00135, p < 0.001, p = 0.01 respectively), but interaction terms (sampling date × treatment) were not significant (all p > 0.05).

**Figure 5:**
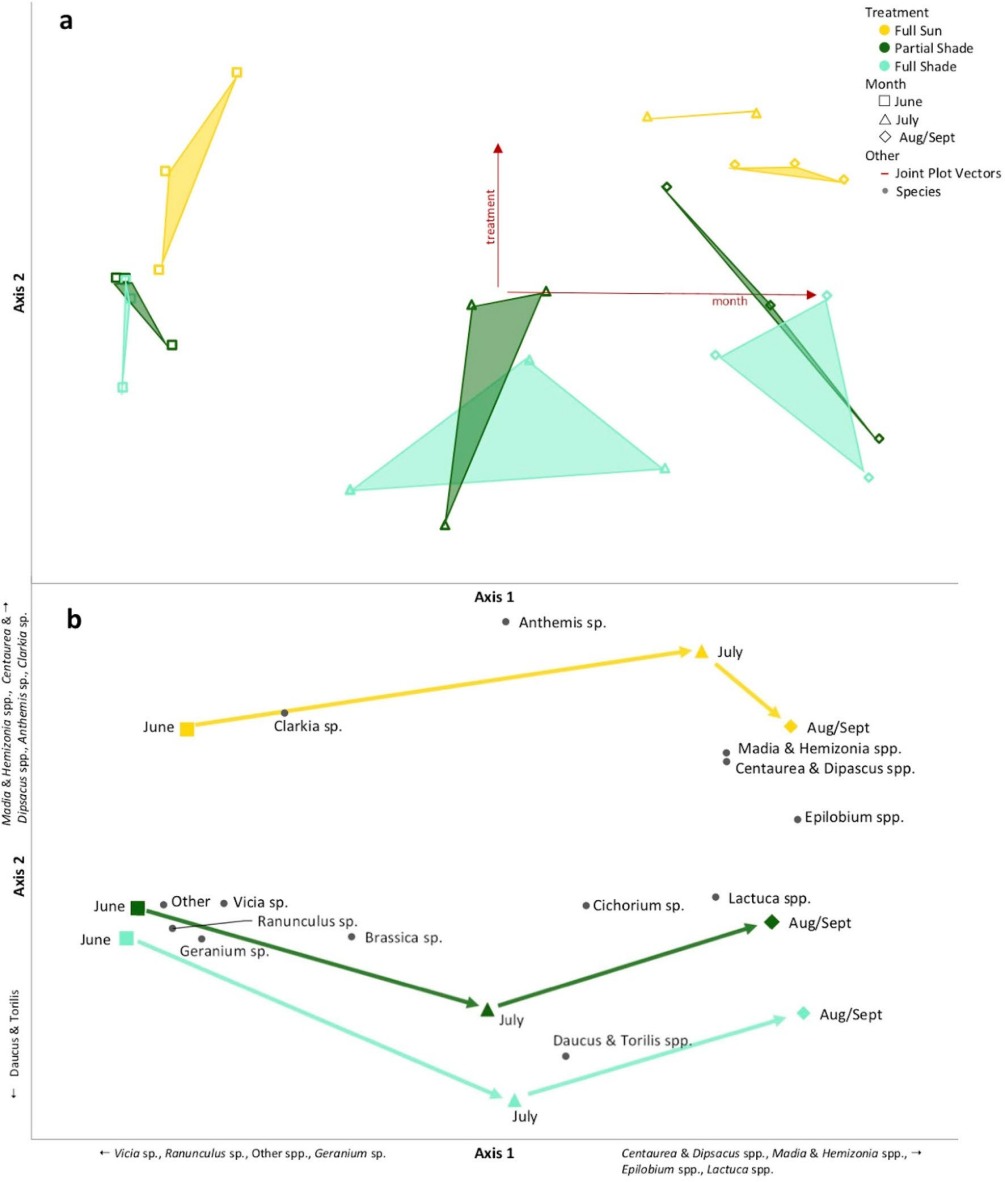
(**a**) Plant community composition described through a nonmetric multidimensional scaling of sample units (averaged by month) in insect species space, with weighted average positions shown for species significantly correlated with axes. Sample units that are close together in the graph are more similar (in species composition) than those that are farther apart. Convex hulls connect groups of treatments (by month). Colored, un-filled symbols represent sample units. Black circles represent species. Joint-plot vectors (red lines) show environmental variables correlated with the axes, vector length represents correlation strength. (**b**) Successional vectors connect centroids from each group of treatments (by month) to illustrate community change over time. Colored, filled symbols represent centroids. Black circles represent species. Fundamental coordinates were generated and data exploration was conducted in PC-ORD Software version 7.07^47^. Figure linework and aesthetics were created in Microsoft PowerPoint 2016.

The NMS ordination of sample units in plant species space produced a two-dimensional solution (final stress = 9.4, final instability = 0, p = 0.004, cumulative R^2^ = 0.87) shown in Fig. 5a,b. Axis 1 described 76.5% of variation, axis 2 described 10.6%. Centroids for each treatment for each month are shown in Fig. 5b. MRPP described significant differences in plant community composition by month (A = 0.48, p < 0.001), but not treatment (A = 0.02, p = 0.27). Vetch (*Vicia* sp.), buttercup (*Ranunculus* sp.), geranium (*Geranium* sp.*)*, and other sp. (*Amsinckia* sp., *Castilleja* sp., *Achyrachaena* sp., etc.) were negatively correlated with axis 1, implying an association with plots sampled in earlier months. Thistles (*Centaurea* sp., *Dipsacus* sp.), tarweed (*Madia* spp., *Hemizonia* sp.), willowherb (*Epilobium* spp.) and lettuce (*Lactuca* spp.) were positively correlated with axis 1, indicative of plots sampled in later months. Carrots (*Daucus* sp., *Torilis* sp.) were also negatively correlated with axis 2, indicating association with shadier treatments. Tarweed (*Madia* spp., *Hemizonia* sp.), thistle (*Centaurea* sp., *Dipsacus* sp.), chamomile (*Anthemis* sp.), and clarkia (*Clarkia* sp.) were positively correlated with axis 2, indicative of sunnier treatments. Correlations with axes are available in Supplementary Material.

Treatment centroids were closest in June (indicating similarity), then diverged in July (indicating dissimilarity), only to reconverge in August/September. In July, the full sun centroid was close to the full sun August/September centroid, indicating similar species composition. Meanwhile the July centroids for partial shade and full shade were distant from the August/September centroids, indicating dissimilarity. This illustrates that full sun plots transitioned to the late-summer plant community, characterized by *Madia* sp, *Hemizonia* sp., *Lactuca* sp., and *Epilobium* sp., before full shade or partial shade plots, indicating a delay in bloom timing.

### Pollinating insects

**Figure 6:**
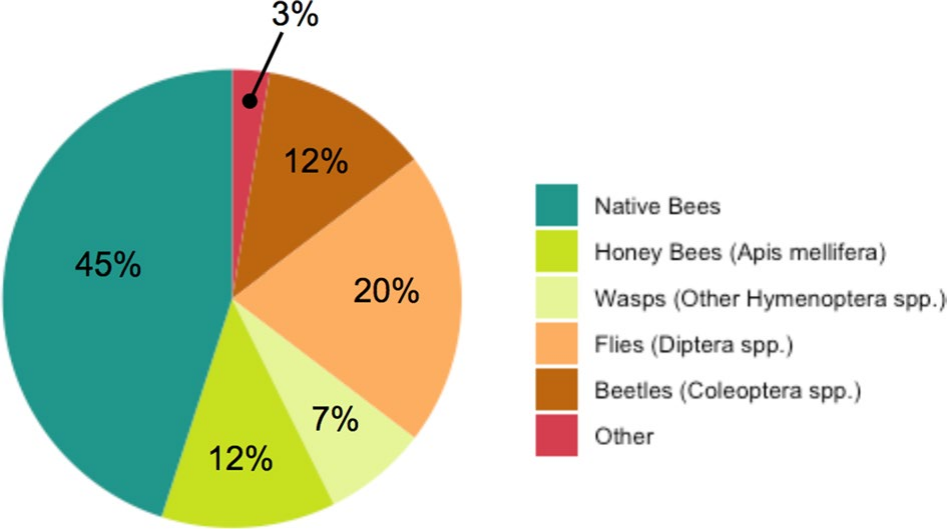
Percentage of pollinating insects contributed by different taxonomic groups, indicated by color.

We collected 342 pollinating insects over the course of the study, representing 65 different insect species. Of these individuals, 45% were native bees, 20% were flies (Diptera spp.), 12% were honey bees (*Apis mellifera*), 12% were beetles (Coleoptera spp.), and 7% were wasps (other Hymenoptera spp.), and 3% were from other taxonomic groups (Lepidoptera, Hemiptera; Fig. 6). The native bee speciemns represented 20 different species, including species from the genera *Bombus* (bumble bee), *Ceratina* (small carpenter bee), *Eucera*, (longhorn bee) *Halictus* (sweat bee), *Lasioglossum* (sweat bee), *Megachile* (leafcutter bee), *Melissodes* (longhorn bee), and *Osmia* (mason bee). We found an average of 3% more pollinating insects per 100 m^2^ in partial shade and full sun plots than in full shade plots (p < 0.001, p < 0.001 respectively, Fig. 4b). Insect species richness was higher in partial shade and full sun than in full shade (p < 0.001, p < 0.001 respectively; Fig. 4c), as was species diversity (p = 0.001, p < 0.001 respectively; Fig. 4d). Species diversity also varied by time (p = 0.011) though interaction terms were not significant. Insect to flower visitation rates did not differ between treatment plots at this scale (p = 0.184).

**Figure 7:**
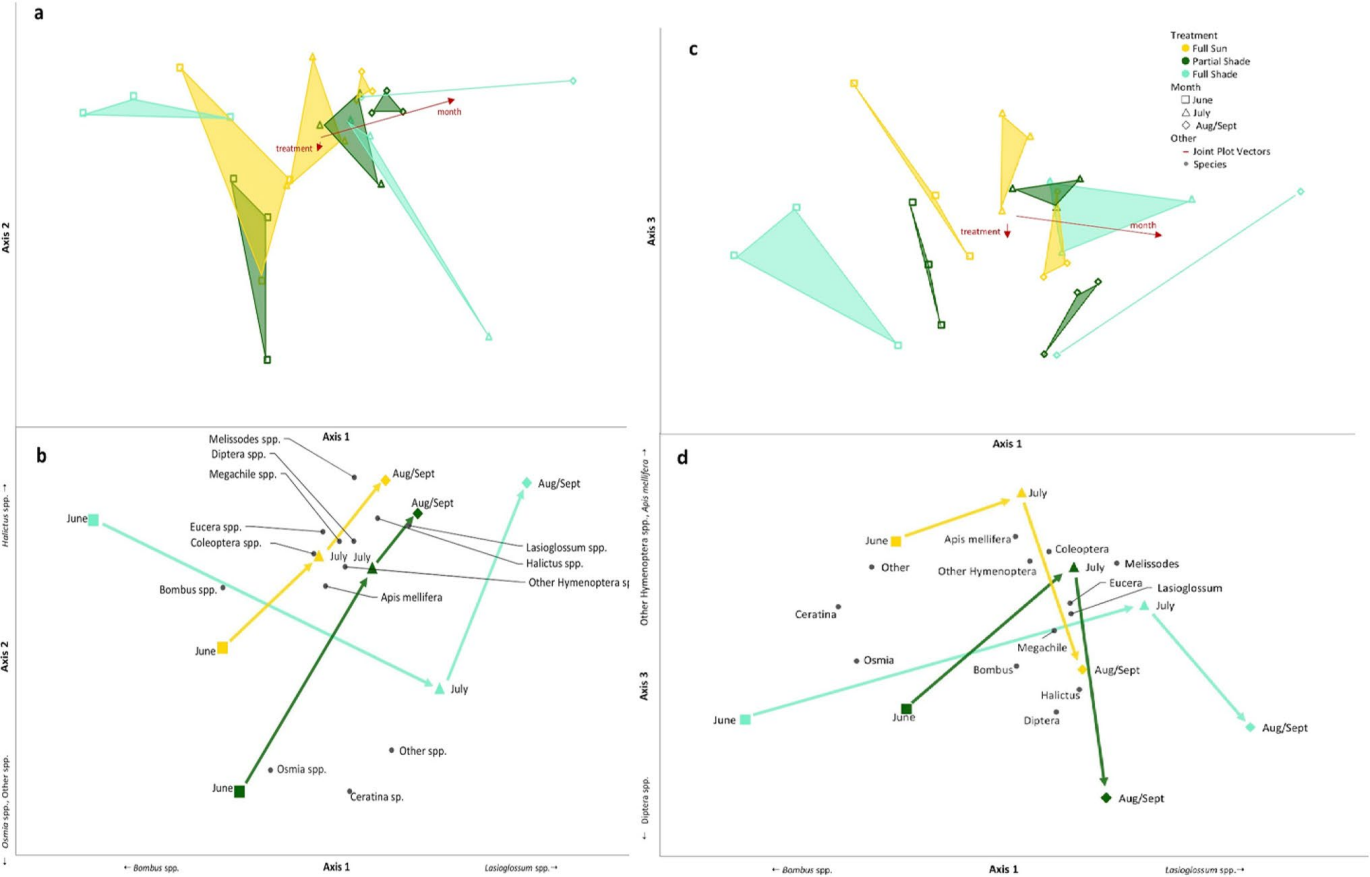
Insect community composition described through a nonmetric multidimensional scaling of sample units (averaged by month) in insect species space, with weighted average positions shown for species significantly correlated with (**a**,**b**) axes 1 and 2 and (**c**,**d**) axes 1 and 3. Sample units that are close together in the graph are more similar (in species composition) than those far apart. (**a**,**c**) Convex hulls connect groups of treatments (by month). Colored, un-filled symbols represent sample units. Black circles represent species. Joint-plot vectors (red lines) show environmental variables correlated with the axes, vector length represents correlation strength. (**b**,**d**) Successional vectors connect centroids from each group of treatments (by month) to illustrate community change over time. Colored, filled symbols represent centroids. Black circles represent species. Fundamental coordinates were generated and data exploration was conducted in PC-ORD Software version 7.07^47^. Figure linework and aesthetics were created in Microsoft PowerPoint 2016.

The NMS ordination of sample units in insect species space produced a three-dimensional solution (final stress = 7.4, final instability = 0, p = 0.012, cumulative R^2^ = 0.85) shown in Fig. 7a–d. Axis 1 described 42% of variation, axis 2 described 21%, and axis 3 described 22%. Centroids for each treatment for each month are shown in Fig. 7b. MRPP described significant differences in community composition by month (A = 0.24, p < 0.001), but not treatment (A = 0.034, p = 0.13). Examination of axes 1 and 2 shows *Bombus* spp., *Osmia* spp., and other spp. (Hemiptera, Lepidoptera) were more common in plots sampled in June, particularly in the partial shade. *Halictus* spp. and *Lasioglossum* spp. were common in plots sampled in July, August, and September (Fig. 7a,b). On axis 2 and 3, we see that *Bombus* spp. and Diptera spp. were common in the full shade and partial shade during June, while *Apis mellifera* and wasps were characteristic of full sun plots in June and July (Fig. 7c,d). Correlations of taxonomic groups with axes are available in the Supplementary Material. Along axes 1 and 2, the centroids for full sun and partial shade plots followed a similar trajectory through insect space, and become closer (more similar) as time progressed. In contrast, the centroids for full shade followed a different trajectory and are farther away from the full sun and partial shade plots, indicating dissimilarity (Fig. 7a,b). Along axis 3, partial shade plots appear more similar to full shade than to full sun. All three treatments are characterized by *Apis mellifera* in July, the move to communities with more Diptera spp. in August and September (Fig. 7c,d).

## Discussion

The differences in floral abundance, and delay in bloom timing that we observed among treatments in this experiment demonstrate that microclimates created by solar panel shading impact plant physiology and morphology, and shed light on how plants might respond to partial shade conditions under solar panels during times of drought. Other researchers have documented changes in plant phenology due to solar panel microclimates in dryland ecosystems at sites with different climates, panel arrangements, and local soil conditions. Adeh et al. ^27^ found elevated biomass of forage grasses in full shade microclimates^27^. Hernandez et al. ^26^ documented increased seedset of desert annuals and perennials in full shade microclimates^26^. What aspects of plant phenology change, and how they change, may depend on individual plant preferences for temperature, moisture, and sunlight.

Angiosperms have evolved strategies to alter bloom time, length and intensity in response to environmental conditions (light, temperature, moisture)^9,48–52^. The effects of shading on flowering depend on the individual species preferences and local growing conditions. Zhao et al.^51^ found that growing herbaceous peony (*Paeonia lactiflora*) flowers in a shaded environment caused declines in key sugars and proteins, which delayed and prolonged flowering^51^. They also observed a decrease in fresh flower weight and an increase in flower diameter, indicating a change in resource allocation as a result of shading^51^. When examining shaded and unshaded coffee plantations, Prado et al.^52^ also observed differences in flower morphology, but did not see a difference in nectar or pollen levels, which are key drivers of pollinating insect populations^52^. The increased floral abundance and delayed bloom timing that we observed in the partial shade (versus full sun) could be the result of reduced sun hours on photoperiodicity^48,49^, photosynthetic efficiency^51^, or transpiration efficiency^53^; the decrease in soil temperature and moisture on germination^49^, root establishment^9^; or a combination of these strategies and mechanisms. Thus when planting solar arrays with flowering plants, land managers may expect to see differences in bloom timing and abundance along shade gradients. At our site, partial shading by solar panels increased bloom abundance by delaying bloom timing, increasing forage for pollinators during the hot, dry, late-season—a time when nutrition is particularly important. Which area (ex. full shade, partial shade, full sun) produces the most blooms may vary based on climate, panel design, and local site conditions.

We observed differences in the abundance, richness, diversity of the pollinator community along shade variations within the solar array, but the lack of a significant correlation between treatment and the ordination axes indicates that there was too much variation in the data to draw conclusions about species specific trends with regard to treatment. Differences in shading may facilitate niche-partitioning as a result of species tolerances for shade, temperature, and floral preference, but more study is needed to show which treatments favor particular insect taxonomic groups.

Since visitation rates did not differ among treatments, but floral abundance did differ among treatments, variations in the pollinator community can be partially attributed to the high variation in the plant community documented at this scale. There may be additional environmental or biological factors (e.g. temperature, wind, pests) impacting the pollinator community within treatments. While we measured temperature before each survey, our measurements were not a scale fine enough to make inferences. Generally, pollinators prefer foraging in sunny rather than shady conditions^34^, although shadier regions may be preferred by some taxa (ex. bumblebees, flies) that have the capacity to forage at lower temperatures^54^. While full sun and partial shade plots were surveyed when plots were sunny, this was not possible in full shade plots, which were actively shaded at the time of survey. Thus active shading likely resulted in lower ambient air temperatures, which could have affected pollinator populations in addition to variations attributed to shade-grown flowers. Even though abundance, richness and diversity were less in full shade than in either partial shade or full shade plots, we still observed pollinators foraging on flowers, and visitation rates were not statistically different at this scale. Future studies may want examine whether pollinators use shade corridors as flyways in addition to pollen and nectar foraging.

Our unreplicated climate observations from partial shade and full sun plots were generally consistent with observations by Barron-Gafford et al.^29^, Adeh et al.^27^, and Marrou et al.^28^, though the magnitude of these measurements varies with panel arrangement, latitude, and time of year. Unfortunately, we are not able to compare our full shade measurements due to possible equipment issues. Replication of all climatic measurements would have improved our ability to interpret these observations, but was outside the scope of this study.Whether or not microclimatic variations are beneficial to plant and insect populations depends largely on specific plant characteristics and local climate. Whether the ecosystem is water-limited (dryland) or light-limited (surplus of water) may influence how plants react to partial shading by solar panels.

When properly sited, pollinator-focused solar provides an opportunity for solar energy development to benefit rather than degrade biodiversity, as has been documented in developments in wildlands of southern California^19^. When placed in areas with high ecosystem services, solar development can negatively impact ecosystem services such as biodiversity^19^. When placed in areas of low ecosystem services, pollinator-focused solar has the potential to positively impact ecosystem services such as biodiversity, and pollination services through the creation of pollinator habitat and restoration of native plants species ^22,24,55^. Agricultural areas in themselves can also promote high biodiversity^56^, so land use tradeoffs should be evaluated on a case-by-case basis.

Additionally, increases in pollinator biodiversity near agricultural lands could increase pollination services to agriculture, which has the potential to increase crop yields and profits^22^. Whether pollinator habitat collocated with solar is more biodiverse or provides more pollination services than pollinator habitat not collocated with solar is unknown, and provides another avenue for future study. Future research should examine the impacts of solar, biodiversity, and agricultural yields on a landscape scale to determine whether benefits are realized.

### Inferences

Observations of species-based performance (ex. which treatment produced the most flowers and of what composition) are not transferable to sites with differing climates, species mixes, and panel arrangements; however, the general trends of the data—that plant communities vary with shade—are consistent with physical mechanisms and prior botanical studies^9,32,48,49,53^. Thus, we can expect both plant and pollinators communities to vary along shading gradients throughout solar arrays, and pollinators to visit flowers despite their proximity to solar panels. We expect that visitation rate will not differ and that floral abundance, bloom timing, insect abundance, insect richness, and insect diversity may vary, following characteristics of the local climate, species mix, and solar panel design.

## Conclusion

Our results show that (1) pollinating insects visited flowers regardless of the presence of solar panels, and (2) that shading from solar panels altered the abundance and timing of floral blooms visited by pollinators, and influenced the abundance, richness and diversity of the pollinator community. Thus, planting solar arrays with pollen and nectar producing plants (flowers) creates habitat for pollinating insects, and "pollinator-friendly" solar installations should include multiple plant species that are shade-tolerant or thrive in full sun to maximize the niche-partitioning inherent in insect pollinator communities. Microclimates with partial shading may provide additional benefits in drylands during hot, dry summers. Unused or underutilized lands below solar panels represent an opportunity to augment current paucity and expected decline of pollinator habitat. Near agricultural lands, this also has the potential to benefit the surrounding agricultural community. Solar developers, policy makers, agricultural communities and pollinator health advocates looking to maximize land use efficiency, biodiversity, and pollination services may consider pollinator habitat at solar photovoltaic sites a viable pathway, while evaluating specific considerations, such as local climate and current land-use, on a case-by-case basis.

## Supplementary Information

Supplementary Information.
