# Supplementary material for: Partial shading by solar panels delays bloom, increases floral abundance during the late-season for pollinators in a dryland, agrivoltaic ecosystem
Source: Sci Rep. 2021 Apr 2;11:7452. doi: 10.1038/s41598-021-86756-4 (PMC8018959; doi:10.1038/s41598-021-86756-4)
Supplement: Supplementary file 1 — Supplementary Information. [file 41598_2021_86756_MOESM1_ESM.docx]

**Supplementary Material**

**Title:** Partial shading by solar panels delays bloom, increases floral abundance during the late-season for pollinators in a dryland, agrivoltaic ecosystem

**Authors:** Maggie Graham*^a^, Serkan Ates,^b^ Andony P. Melathopoulos^c^, Andrew R. Moldenke^d^, Sandra J. DeBano^e^, Lincoln R. Best^c^, Chad W. Higgins^a^

*Correspondence to grahaann@oregonstate.edu

^a^Department of Biological and Ecological Engineering, Oregon State University, Corvallis, OR 97330, USA.

^b^Department of Animal and Rangeland Sciences, Oregon State University, Corvallis, OR 97330, USA.

^c^Department of Horticulture, Oregon State University, Corvallis, OR 97330, USA.

^d^Department of Botany and Plant Pathology, Oregon State University, Corvallis, OR 97330, USA.

^e^Department of Fisheries and Wildlife, Oregon State University, Hermiston Agricultural Research and Extension Center, Hermiston, Oregon 97838, USA.

**Author Contributions:** MG, SA, APM, and CWH developed ideas, designed methodology. MG, SA, and SJD conducted statistical analysis. ARM, LRB, and MG identified insect specimens. MG established research sites, collected data, and led writing of the manuscript. All authors reviewed the results and contributed to the writing of the manuscript.

**Supplementary Material**

Table of Contents SI-2

Supplementary Figure S1 SI-3

Supplementary Figure S2 SI-4

Appendix A. Restoration Seed Mix SI-5

Appendix B. Code SI-6

Appendix C. Plant Species and Grouping SI-7

Appendix D. Insect Species and Grouping SI-8

Appendix E. Pearson Correlations SI-10

**
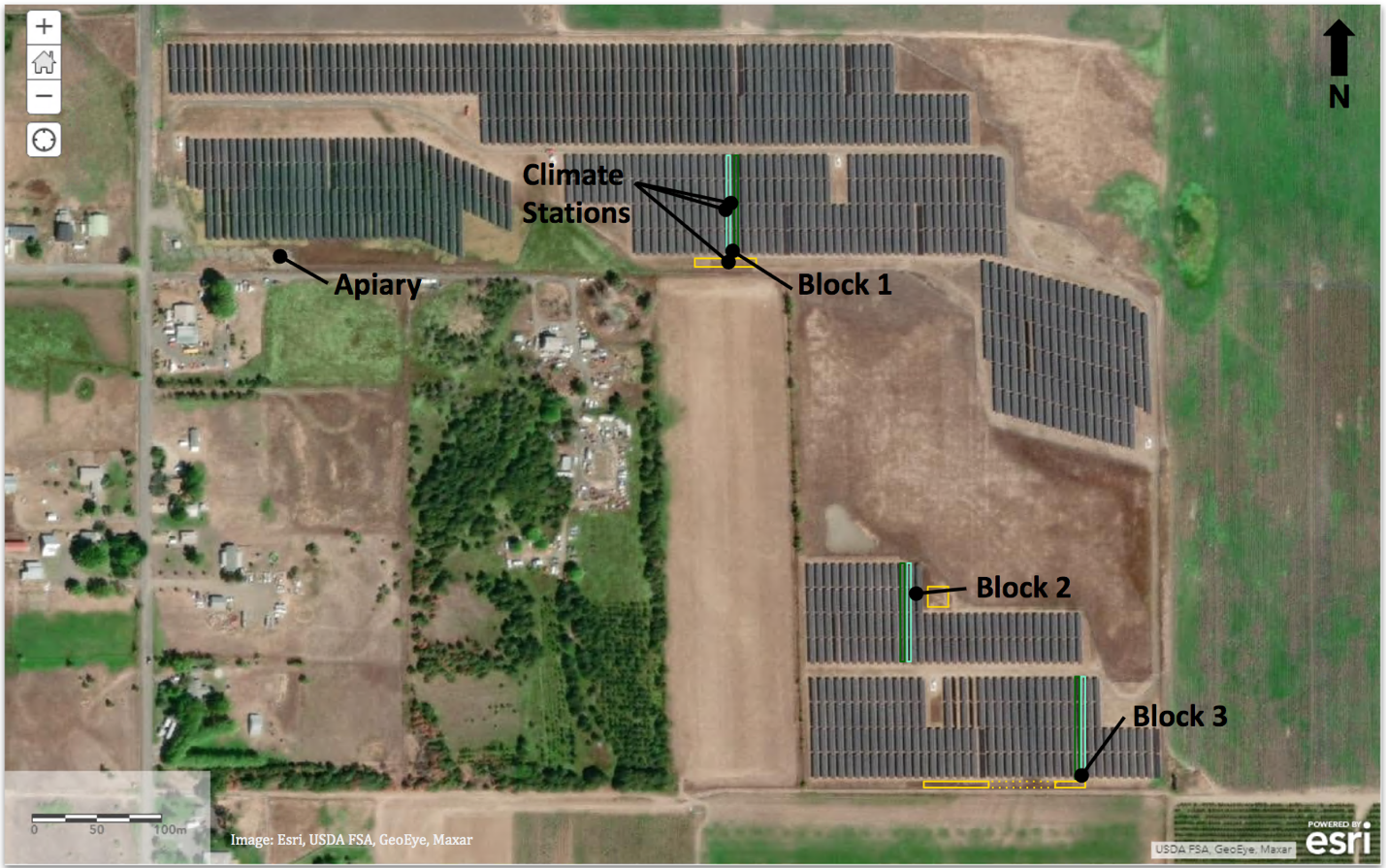
**

Figure S1. Block locations within the Eagle Point Solar Plant. We established a randomized complete block design with three replicates, each with three treatments. Climate stations were positioned in Block 1. An apiary is located along the western edge of the site, within flight distance of all survey locations. Base Imagery Source: Esri 2021, USDA FSA, GeoEye, Maxar.


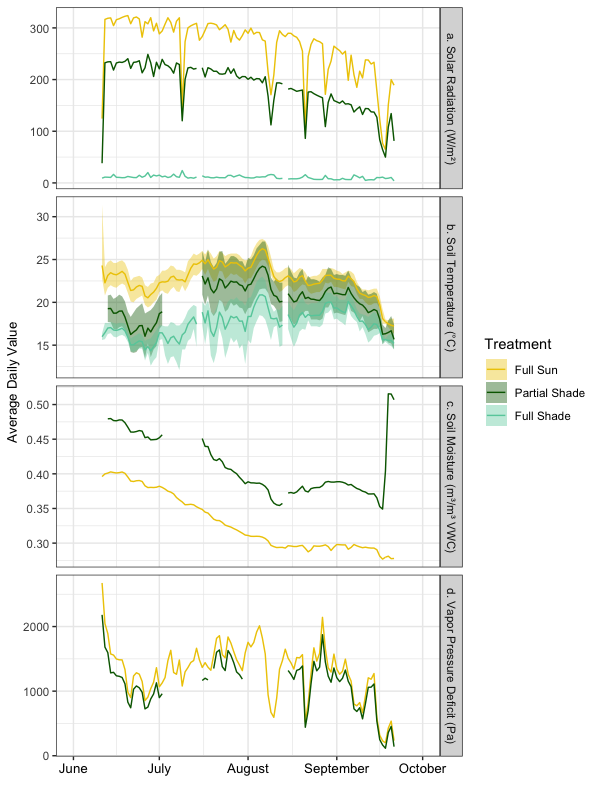


Figure S2: Daily averages of microclimatic variables by measurement type: (a) Solar radiation, (b) soil temperature with shaded regions showing the daily high/low, (c) soil moisture, and (d) vapor pressure deficit (VPD). Indicated by different color for three treatments: (1) full sun (2) partial shade, and (3) full shade. VPD was not measured in the full shade, and soil moisture in the full shade is not reproduced because of a possible sensor error.

**Appendix A: Restoration Seed Mix**

Restoration seed mix provided courtesy of Lomakatsi Restoration Project^40^

Species Type Amount (lbs)

*Amsinckia menziesii* Annual forb 3

*Clarkia purpurea* Annual forb 12

*Collinsia grandiflora* Annual forb 5

*Daucus pusillus* Annual forb 10

*Gillia capitata*  Annual forb 7.5

*Hemizonia fitchii*  Annual forb 0.5

*Helianthus bolanderi*  Annual forb 0.5

*Lupinus bicolor*  Annual forb 0.5

*Madia elegans*  Annual forb 3

*Navarretia intertextata* Annual forb 1

*Plagiobothrys figuratus*  Annual forb 7

*Festuca roemeri* Grass 130

*Carex densa*  Sedge 6

*Carex pachystachya* Sedge 4

*Carex tumulicola* Sedge 4

*Juncus tenuis*  Rush 0.5

*Achillea millefolium* Perennial forb 5

*Agoseris grandiflora* Perennial forb 3

*Asclepias fascicularis*  Perennial forb 10

*Camassia quamash* Perennial forb 1

*Cynoglossum grande* Perennial forb 0.5

*Eriophyllum lanatum* Perennial forb 18

*Grindelia nana*  Perennial forb 20

*Lomatium utriculatum* Perennial forb 0.5

*Lupinus microcarpus* Perennial forb 5

*Lupinus adsurgens*  Perennial forb 5

*Ranunculus austrooreganus* Perennial forb 0.5

*Wyethia angustifolia*  Perennial forb 1

**Appendix B. Code**

##This file provides calculations of repeated measures ANOVA and paired t-test used in data analysis.

##Import data and packages

library(ggplot2)

polsol <- read.csv("~/Desktop/R_files/Overview_2.19.21.csv", header=TRUE)

View(polsol)

##Set time as a factor

polsol$time <- as.factor(polsol$time)

#class(polsol$time)

##Select data for analysis

#polsol$testdata <-polsol$visitationrate

#polsol$testdata <-polsol$log10blooms

#polsol$testdata <-polsol$log10insects

#polsol$testdata <-polsol$richness_bl

#polsol$testdata <-polsol$richness_ins

#polsol$testdata <-polsol$diversity_shan_bl

#polsol$testdata <-polsol$diversity_shan_ins

##Check Assumptions

##(1)Normal distribution? (histogram, q-q plot)

qplot(testdata, data=polsol, geom="histogram", bins = 25, main = "Distribution of Data") +

facet_grid(treatment ~ .)

qqnorm(polsol$testdata)

qqline(polsol$testdata, col = "steelblue", lwd = 2)

##(2)Equal std deviation? (boxplot)

ggplot(data=polsol, aes(x=treatment, y=testdata)) +

geom_boxplot() +

stat_summary(fun.y=mean, geom="point", shape=3, size=3)+

theme_bw()+

labs(title = "Standard Deviation of Data", legend = "Treatment")

##Are there differences?

##One-factor ANOVA with repeated measures (time), with variable selected above.

rm.aov <- aov(testdata~Error(replicate)+treatment*time, data=polsol)

summary(rm.aov)

##Which treatments are different?

##Paired t-test

t.test(formula = testdata ~ treatment,

data = polsol,

subset = treatment %in% c( 'Full Sun', 'Partial Shade'),paired=TRUE, var.equal=TRUE)

t.test(formula = testdata ~ treatment,

data = polsol,

subset = treatment %in% c('Full Sun', 'Full Shade'),paired=TRUE, var.equal=TRUE)

t.test(formula = testdata ~ treatment,

data = polsol,

subset = treatment %in% c('Partial Shade', 'Full Shade'), paired=TRUE, var.equal=TRUE)

##Bonferroni correction

pairwise.t.test(polsol$testdata, polsol$treatment, p.adj = 'bonferroni')

##Calculate means and sd for each treatment

aggregate(testdata~treatment, data=polsol, FUN=mean)

aggregate(testdata~treatment, data=polsol, FUN=sd)

**Appendix C: Plant Species and Grouping**

| Group Name | Plant Species Name |
| --- | --- |
| *Anthemis* sp. (Chamomile) | *Anthemis_cotula* |
| *Brassica* sp. (Mustards) | *Brassica_nigra* |
| *Centaurea* & *Dipsacus* spp. (Thistles) | *Centaurea_solstitialis* |
| *Centaurea* & *Dipsacus* spp. (Thistles) | *Dipsacus_fullonum* |
| *Cichorium* sp. (Chichory) | *Cichorium_intybus* |
| *Clarkia* sp. (Clarkia) | *Clarkia_purpurea* |
| *Daucus* & *Torilis* sp. (Carrot) | *Daucus_carota* |
| *Daucus* & *Torilis* sp. (Carrot) | *Torilis_arvensis* |
| *Epilobium* spp. (Willowherbs) | *Epilobium_densiflorum* |
| *Epilobium* spp. (Willowherbs) | *Epilobium_minutum* |
| *Geranium* sp. (Geranium) | *Geranium_dissectum* |
| *Hemizonia* & *Madia* spp. (Tarweeds) | *Hemizonia_congesta* |
| *Hemizonia* & *Madia* spp. (Tarweeds) | *Madia_elegans* |
| *Hemizonia* & *Madia* spp. (Tarweeds) | *Madia_gracilis* |
| *Lactuca* spp. (Lettuces) | *Lactuca_serriola* |
| *Lactuca* spp. (Lettuces) | *Lactuca*_sp. |
| Other spp. | *Achyrachaena_mollis* |
| Other spp. | *Amsinckia_menziesii* |
| Other spp. | *Brodiaea_elegans* |
| Other spp. | *Castilleja_tenuis* |
| Other spp. | *Cerastium_fontanum* |
| Other spp. | *Medicago_lupulina* |
| Other spp. | *Valerianella_locusta* |
| Other spp. | *Veronica_americana* |
| *Ranunculus* sp. (Buttercup) | *Ranunculus*_sp. |
| *Vicia* sp. (Vetch) | *Vicia_americana* |

**Appendix D: Insect Species and Grouping**

| Group Name | Insect Species Name |
| --- | --- |
| *Apis mellifera* (Honey Bee) | *Apis_mellifera* |
| *Bombus* spp. (Bumblebee) | *Bombus_caliginosus* |
| *Bombus* spp. (Bumblebee) | *Bombus_fervidus* |
| *Bombus* spp. (Bumblebee) | *Bombus_griseocollis* |
| *Bombus* spp. (Bumblebee) | *Bombus_nevadensis* |
| *Bombus* spp. (Bumblebee) | *Bombus_rufocinctus* |
| *Bombus* spp. (Bumblebee) | *Bombus_vosnesenskii* |
| *Ceratina* sp. (Small Carpenter Bee) | *Ceratina_acantha* |
| Coleoptera spp. (Beetle) | *Diabrotica_undecimpunctata* |
| Coleoptera spp. (Beetle) | *Trichodes_ornatus* |
| Coleoptera spp. (Beetle) | *Coccinella_septempunctata* |
| Coleoptera spp. (Beetle) | *Coccinella*_sp. |
| Coleoptera spp. (Beetle) | *Hippodamia_convergens* |
| Coleoptera spp. (Beetle) | *Epicauta_puncticollis* |
| Coleoptera spp. (Beetle) | *Chrysochus_cobaltinus* |
| Diptera spp. (Fly) | *Lepidanthrax*_sp. |
| Diptera spp. (Fly) | *Toxophora*_sp. |
| Diptera spp. (Fly) | *Villa_lateralis* |
| Diptera spp. (Fly) | *Eristalis_hirtus* |
| Diptera spp. (Fly) | *Eupeodes_volucris* |
| Diptera spp. (Fly) | *Helophilus_fasciatus* |
| Diptera spp. (Fly) | *Melanostoma_mellinum* |
| Diptera spp. (Fly) | *Paragus*_sp.1 |
| Diptera spp. (Fly) | *Scaeva_pyrastri* |
| Diptera spp. (Fly) | *Sphaerophoria_sulphuripes* |
| Diptera spp. (Fly) | *Syritta_pipiens* |
| Diptera spp. (Fly) | *Syrphus_opinator* |
| Diptera spp. (Fly) | *Toxomerus_marginatus* |
| Diptera spp. (Fly) | *Toxomerus_occidentalis* |
| Diptera spp. (Fly) | *Tachinidae*_sp.1 |
| Diptera spp. (Fly) | *Tachinidae*_sp.2 |
| Diptera spp. (Fly) | *Tachinidae*_sp.3 |
| Diptera spp. (Fly) | *Tachinidae*_sp.4 |
| Diptera spp. (Fly) | *Tachinidae*_sp.5 |
| *Eucera* sp. (Longhorn Bee) | *Eucera*_sp.1 |
| *Halictus* spp. (Sweat Bee) | *Halictus_ligatus* |
| *Halictus* spp. (Sweat Bee) | *Halictus_tripartitus* |
|  |  |
| Group Name | **Insect Species Name** |
| *Lasioglossum* spp. (Sweat Bee) | *Lasioglossum*_sp. |
| *Lasioglossum* spp. (Sweat Bee) | *Lasioglossum_titusi* |
| *Megachile* spp. (Leafcutter Bee) | *Megachile_apicalis* |
| *Megachile* spp. (Leafcutter Bee) | *Megachile_brevis* |
| *Megachile* spp. (Leafcutter Bee) | *Megachile*_sp. 1 |
| *Megachile* spp. (Leafcutter Bee) | *Megachile_perihirta* |
| *Melissodes* spp. (Longhorn Bee) | *Melissodes_lupina* |
| *Melissodes* spp. (Longhorn Bee) | *Melissodes_metenuus* |
| *Melissodes* spp. (Longhorn Bee) | *Melissodes_pallidisignata* |
| *Osmia* spp. (Mason Bee) | *Osmia_nemoris* |
| Other Hymenoptera spp. (Wasp) | *Steniolia_scolopacea* |
| Other Hymenoptera spp. (Wasp) | *Ichneumonidae*_sp.1 |
| Other Hymenoptera spp. (Wasp) | *Ichneumonidae*_sp.2 |
| Other Hymenoptera spp. (Wasp) | *Ichneumonidae*_sp.3 |
| Other Hymenoptera spp. (Wasp) | *Ichneumonidae*_sp.4 |
| Other Hymenoptera spp. (Wasp) | *Cryptocheilus*_sp.1 |
| Other Hymenoptera spp. (Wasp) | *Sphecidae*_sp.1 |
| Other Hymenoptera spp. (Wasp) | *Sphecidae*_sp.2 |
| Other Hymenoptera spp. (Wasp) | *Sphecidae*_sp.3 |
| Other Hymenoptera spp. (Wasp) | *Polistes_fuscatus* |
| Other Hymenoptera spp. (Wasp) | *Vespula_vulgaris* |
| Other spp. | *Jalysus*_sp. |
| Other spp. | *Boisea_rubrolineata* |
| Other spp. | *Coenonympha_tullia* |
| Other spp. | *Pieris_rapae* |

**Appendix E: Pearson Correlations**

1. Plant Species: Pearson Correlations with Ordination Axes N= 26

| **Axis:** | **1** | **2** |
| --- | --- | --- |
| **Group Name** | **r** | **r** |
| Other spp. | -0.751 | -0.027 |
| Anthemis sp. | 0.012 | 0.473 |
| Brassica spp. | -0.144 | -0.065 |
| Centaurea & Dipsacus spp. | 0.505 | 0.495 |
| Cichorium sp. | 0.169 | -0.024 |
| Clarkia sp. | -0.303 | 0.444 |
| Daucus & Torilis spp. | 0.15 | -0.559 |
| Epilobium spp. | 0.529 | 0.228 |
| Geranium sp. | -0.623 | -0.145 |
| Madia & Hemizonia spp. | 0.507 | 0.529 |
| Lactuca spp. | 0.876 | 0.005 |
| Ranunculus sp. | -0.816 | -0.128 |
| Vicia sp. | -0.917 | -0.031 |

1. Insect Species: Pearson Correlations with Ordination Axes N= 26

| Axis: | 1 | 2 | 3 |
| --- | --- | --- | --- |
| Group Name: | **r** | **r** | **r** |
| Apis mellifera | 0.009 | -0.003 | -0.425 |
| Other spp. | -0.242 | 0.575 | -0.265 |
| Bombus spp. | 0.509 | 0.003 | 0.151 |
| Ceratina sp. | -0.045 | 0.392 | -0.068 |
| Coleoptera spp. | 0.09 | -0.2 | -0.6 |
| Other Hymenoptera spp. | -0.091 | -0.098 | -0.422 |
| Diptera spp. | -0.228 | -0.373 | 0.586 |
| Eucera spp. | 0.012 | -0.132 | -0.096 |
| Halictus spp. | -0.364 | -0.467 | 0.312 |
| Lasioglossum spp. | -0.539 | -0.38 | -0.226 |
| Megachile spp. | -0.048 | -0.175 | -0.06 |
| Melissodes spp. | -0.081 | -0.31 | -0.25 |
| Osmia spp. | 0.23 | 0.686 | 0.112 |
